# Blood Glucose Fluctuations in Type 2 Diabetes Patients Treated with Multiple Daily Injections

**DOI:** 10.1155/2016/1028945

**Published:** 2015-12-29

**Authors:** Feng-fei Li, Li-yuan Fu, Wen-li Zhang, Xiao-fei Su, Jin-dan Wu, Jin Sun, Lei Ye, Jian-hua Ma

**Affiliations:** ^1^Department of Endocrinology, Nanjing First Hospital, Nanjing Medical University, Nanjing 210012, China; ^2^Nanjing University of Chinese Medicine, Nanjing 210023, China; ^3^National Heart Research Institute Singapore, National Heart Centre Singapore, Singapore 169606

## Abstract

To compare blood glucose fluctuations in type 2 diabetes mellitus (T2DM) patients were treated using three procedures: insulin intensive therapy which is continuous subcutaneous insulin infusion (CSII), MDI3 (three injections daily), and MDI4 (four injections daily). T2DM patients were hospitalized and were randomly assigned to CSII, aspart 30-based MDI3, and glargine based MDI4. Treatments were maintained for 2-3 weeks after the glycaemic target was reached. After completing the baseline assessment, 6-day continuous glucose monitoring (CGM) was performed before and after completion of insulin treatment. Treatment with CSII provided a greater improvement of blood glucose fluctuations than MDI (MDI3 or MDI4) therapy either in newly diagnosed or in long-standing T2DM patients. In long-standing diabetes patients, the MDI4 treatment group had significantly greater improvement of mean amplitude glycemic excursion (MAGE) than the MDI3 treatment group. However, in patients with newly diagnosed diabetes, there were no significant differences in the improvement of MAGE between MDI3 and MDI4 groups. Glargine based MDI4 therapy provided better glucose fluctuations than aspart 30-based MDI3 therapy, especially in long-standing T2DM patients, if CSII therapy was not available.

## 1. Introduction

Intensive insulin therapy may be necessary if conventional therapies were no longer sufficient to maintain glycemic control in patients with type 2 diabetes mellitus (T2DM) [1]. Intensive insulin therapy consists of continuous subcutaneous insulin infusion (CSII) using an insulin pump and multiple daily injections (MDIs). Several studies have demonstrated that early implementation of a short course of intensive insulin therapy may dramatically improve beta-cell function in most patients with newly diagnosed T2DM. This improvement of *β*-cell function might be responsible for the remission described in newly diagnosed T2DM patients [2–5]. However, the clinical response to short-term CSII may be variable, and this is probably a reflection of the heterogeneity of T2DM. Some have suggested that patients with higher late-phase insulin secretion may be able to benefit most in improvement of beta-cell function with CSII intervention [6]. Very recently, the OpT2mise group confirmed that even patients with long-standing T2DM for many years, despite previous use of MDI, are still able to achieve further significant improvement of the mean glycated haemoglobin (HbA1c) with CSII and with decreased blood glucose fluctuations [7].

CSII has become common practice in the world. Although MDI is inferior in control patient blood sugar levels compared with CSII, many people with T2DM are still struggling to keep their blood glucose values in target range by MDI. MDIs are three or more injections daily with long-acting or short-acting insulin. However, the knowledge of CSII or MDI therapy (three or more injections daily) for T2DM patients favouring better glucose fluctuation control is still limited.

We, therefore, performed a randomised, parallel-group trial using continuous glucose monitoring (CGM) to assess the blood glucose fluctuations in T2DM patients, who achieved euglycemic control treated with two procedures of intensive insulin therapy, that is, MDI, aspart 30-based MDI3 (three injections daily), and glargine based MDI4 (four injections daily).

## 2. Methods

This was a randomised, parallel-group study consisting of a run-in period and a 2- to 3-week randomised phase. Patients with newly diagnosed and long-standing T2DM were enrolled from eight centres in China between February, 2010, and December, 2014. The patients with the age of 18–80 years were required to have HbA1c values ranging from 9.0% to 12.0%. Patients were excluded if they were positive for antiglutamic acid decarboxylase antibodies, pregnant, or planning to become pregnant. Patients with maturity onset diabetes in youth and mitochondria diabetes mellitus, with cognitive disorder, or with abuse of alcohol or drugs were also excluded [8]. There was a 4- to 6-day run-in period of diet alone. The protocol and informed consent document were approved by institutional Ethics Committee approval at each of the study centres. All patients gave written informed consent.

All patients were admitted to hospitals. Fasting blood samples were collected for measuring FPG and insulin in all patients before and after treatment (2 days after insulin cessation). Fasting blood samples were obtained for insulin and C-peptide determination. Continuous glucose monitoring (CGM) data were obtained with Medtronic Minimed CGM Gold (Medtronic Incorporated, Northridge, USA) for at least 6 days before randomization and after treatments, as described in a previous study [9].

After completing the baseline assessment and 3 days of CGM, patients (with newly diagnosed T2DM) were randomly assigned into CSII group (CSII N, hereafter), aspart 30-based, three injections daily, group (MDI3 N, hereafter), and glargine based, four injections daily, group (MDI4 N, hereafter). Long-standing T2DM patients were also randomly assigned into the previously mentioned three groups (CSII L, MDI3 L, and MDI4 L, hereafter). Patients in CSII group were provided with aspart (Novo Nordisk, Bagsvaerd, Denmark) using Medtronic insulin pump (Northridge, CA). Initial insulin doses were calculated as 0.4–0.5 IU/kg and were equally administered as basal and bolus injection. Insulin doses were subsequently adapted by the treating physician according to blood glucose values obtained by self-monitoring. Patients in MDI3 group were injected aspart 30 (Novo Nordisk, Bagsvaerd, Denmark) before each meal. Patients in MDI4 group were injected aspart before each meal and glargine (Sanofi-Aventis Pharmaceuticals, Paris, France) at bedtime. Premeal doses were also calculated as 0.4–0.5 IU/kg and distributed evenly throughout every premeal. Euglycemic control was achieved if the fasting capillary blood glucose was less than 6.1 mmol/L and capillary blood glucose at 2 h after each of three meals was less than 8.0 mmol/L [8]. Investigators titrated insulin doses on an individual-patient basis at the titration algorithm (if the fasting blood glucose level was less than 4.4 mmol/L, the insulin dose was reduced 2 units; if the fasting blood glucose level was within 4.4 to 6.1 mmol/L, the insulin dose was unchanged; if the fasting blood glucose level was within 6.2 to 7.8, within 7.9 to 10.0, and >10.0 mmol/L, the insulin dose was increased subsequently by 2, 4, and 6 units, resp.). When euglycemic control was achieved, treatments were remained unchanged and maintained for 2-3 weeks.

The 24 h mean amplitude of glycemic excursions (MAGE) and other plasma glucose fluctuation parameters such as the 24 h mean blood glucose (MBG), the standard deviation (SD) of the MBG, the percentage time duration (%), and the incremental area under curve (AUC) of plasma glucose >10.0 mmol/L and <3.9 mmol/L was calculated, and hypoglycemia episodes were also recorded. MAGE was calculated for each patient by measuring the arithmetic mean of the ascending and descending excursions between consecutive peaks and nadirs for the same 24 h period; only absolute excursion values >1 SD were considered [10]. HbA1c was measured centrally at the Department of Endocrinology, Nanjing First Hospital, Nanjing Medical University. Radioimmunoassay was used for measurement of insulin (Beijing Technology Company, Beijing, China). 8-iso prostaglandin F_2*α*_ (8-iso PGF_2*α*_) was measured using an enzyme immunoassay method (Cayman Chemical Co., Ann Arbor, MI). Tumor necrosis factor-*α* (TNF-*α*) was measured using the human-specific Milliplex map kit according to the manufacturer's instructions (Millipore, St. Charles, MO, USA). Interleukin-6 (IL-6) was determined using commercially available Enzyme-Linked Immunosorbent Assay kits according to manufacturer's instructions (R&D Systems, Minneapolis, MN, USA). Routine clinical laboratory tests were done in the central laboratory units of the eight participating centres. Basal *β*-cell function and insulin resistance were estimated by homoeostasis model assessment-B (HOMA-B) and (HOMA-IR), which were calculated as previously described [8, 11].

The primary endpoint was the between-group differences of 24 h MAGE. Secondary endpoints were the effect of different interventions on oxidative stress, inflammatory levels, and *β*-cell function in these patients. The MAGE, 24 h MBG, AUC for hypoglycemia (defined as sensor glucose values <3.9 mmol/L) and hyperglycemia (sensor glucose values >10 mmol/L), and time spent in hypoglycemia and hyperglycemia were also analyzed.

### 2.1. Statistical Analysis

Data were analyzed with the SPSS PASW Statistics 18 Package. Normally distributed and continuous variables are presented as mean (standard deviation, SD). Nonnormally distributed variables were presented as median (IQR) and logarithmically transformed before analysis. The independent samples *t*-test was used to compare each group difference. Significance was defined as *P* < 0.05.

This study was registered with Chinese Clinical Trial Registry, number ChiCTR-TRC-11001218.

## 3. Results

Table 1 gives the baseline characteristics of the 116 newly diagnosed patients and the 127 patients with long-standing T2DM patients. The 116 newly diagnosed patients were allocated randomly to the CSII N (39), the MDI3 N (38), and the MDI4 N groups (39); and the 127 long-standing T2DM patients were randomly allocated to the CSII L (43), the MDI3 L (41), and the MDI4 L groups (43). There were no significant demographic differences between the different groups at baseline (Table 1).

### 3.1. The Effects of Transient Intensive Insulin Therapy on Metabolic Control

#### 3.1.1. Glycemic Control

Significant improvement in blood glucose control was achieved in both CSII and MDI groups (fasting capillary blood glucose was <6.1 mmol/L and capillary blood glucose at 2 h after each of three meals was <8.0 mmol/L). Patients in the CSII group reached glycemic goals significantly earlier than in the MDI groups, in the newly diagnosed T2DM patients (4.26 ± 1.88 days in CSII N group, 6.17 ± 2.36 days in MDI3 N group, and 5.81 ± 2.46 days in MDI4 N group; *P* < 0.05 for CSII N group versus MDI3 N group or MDI4 N group; *P* > 0.05 for MDI3 N group versus MDI4 N group) and also in the long-standing T2DM patients (CSII L group 5.45 ± 2.76 days, MDI3 L group 6.39 ± 3.81 days, and MDI4 N group 6.28 ± 2.19 days; *P* < 0.05 for CSII L group versus MDI3 L group or MDI4 L group; *P* > 0.05 for MDI3 L group versus MDI4 L group). In addition, there were no differences in the mean daily insulin doses in all groups (36.91 ± 10.87 IU/day in the CSII N group, 38.45 ± 13.62 IU/day in the MDI3 N group, 37.86 ± 15.90 IU/day in the MDI4 N group, 38.20 ± 17.47 IU/day in the CSII L group, 40.14 ± 18.54 IU/day in the MDI3 L group, and 41.01 ± 20.77 IU/day in the MDI4 L group).

#### 3.1.2. Oxidative Stress and Inflammatory Profile

To determine the effect of transient insulin intensive therapy on oxidative stress, we measured 8-PGF_2*α*_, a well-recognized biomarker of oxidative stress. Compared to baseline, serum 8-PGF_2*α*_ levels were significantly decreased in all groups after transient intensive therapy (Table 2). There was no significant difference between any of the treatment groups.

In order to determine the effect transient intensive insulin therapy on inflammation, we measured serum levels of TNF-*α* and IL-6 reflecting the inflammatory profile in patients with T2DM [12]. Patients in all groups had higher inflammatory cytokine levels at baseline. After transient intensive insulin treatment, we found an improvement of inflammatory cytokines in all groups (*P* < 0.01) (Table 2). CSII and MDI4 therapies had greater decrease of serum levels of IL-6 and TNF-*α* compared to MDI3 therapy in long-standing T2DM patients (*P* < 0.05) (Table 2).

### 3.2. The Effects of Transient Intensive Insulin Therapy on Blood Glucose Fluctuation Control

We collected CGM data at baseline and on 5 days after euglycemic control achieved. The 24 h mean glucose concentrations were significantly decreased after therapy either in newly diagnosed T2DM (Table 3) or in long-standing T2DM patients (Table 4). Of patients who achieved the target glycaemic goals, the 24 h mean glucose concentration was similar in all groups (*P* < 0.01). However, in newly diagnosed T2DM patients, the MAGE in the CSII group was significantly decreased compared to both MDI groups (CSII N group 4.51 ± 1.92 mmol/L, MDI3 N group 5.05 ± 1.97 mmol/L, MDI4 N group 4.94 ± 2.21 mmol/L, *P* < 0.05 versus MDI3 N group and MDI4 N group). There was no statistically significant difference between MDI3 N group and MDI4 N group (*P* > 0.05). The incremental AUC (>10 mmol/L) detected by CGM was not significantly decreased (0.29 ± 0.57 mmol/L per day) in CSII N group compared with the MDI3 N group (0.27 ± 0.37 mmol/L per day) and MDI4 N group (0.27 ± 0.35 mmol/L per day) (*P* > 0.05 versus MDI3 N group and MDI4 N group). The time spent in normal glycaemia (%) (between 3.9 and 10.0 mmol/L) in CSII N group was not significantly increased compared to MDI3 N group and MDI4 N group (85% ± 16 in CSII N group, 83% ± 13 mmol/L per day in MDI3 N group, and 84% ± 15 mmol/L per day in MDI4 N; *P* > 0.05 versus MDI3 N group and MDI4 N group).

Long-standing T2DM patients also achieved significantly better improvement of MAGE in CSII therapy compared with the MDI3 or MDI4 treatment group (CSII L group 4.62 ± 2.97 mmol/L, MDI3 L group 6.27 ± 1.83 mmol/L, and MDI4 L group 5.16 ± 1.98 mmol/L; *P* < 0.01 versus MDI3 L group; *P* < 0.05 versus MDI4 L group). In addition, our results showed that patients in MDI4 L group had significantly improved MAGE compared to patients in MDI3 L treatment group (*P* < 0.05). The incremental AUC (>10 mmol/L) in patients treated with CSII therapy was not significantly different (16% ± 17) compared with MDI3 L group (19% ± 10) and MDI4 L group (18% ± 15) (*P* > 0.05 versus MDI3 L group and MDI4 L group). Again, the time duration in normoglycemia (%) (>3.9 and <10.0 mmol/L) in the CSII L treatment group and that in MDI4 L group were significantly increased compared to the MDI3 L group (83% ± 17 in CSII L group and 79% ± 11 in MDI4 L group, *P* < 0.05 CSII L group and MDI4 L group versus MDI3 L group).

There were no serious hypoglycemic episodes, defined as an event requiring the assistance of another person or other resuscitative treatments, in any treatment group. However, the time spent in hypoglycemia (<3.9 mmol/L) (%) detected by CGM was significantly decreased by the use of transient insulin intensive treatment either in newly diagnosed (Table 3) or in advanced T2DM patients compared with the baseline before treatment (Table 4).

### 3.3. The Effect of Transient Insulin Intensive Therapy on *β*-Cell Function and Insulin Resistance

**Figure 1:**
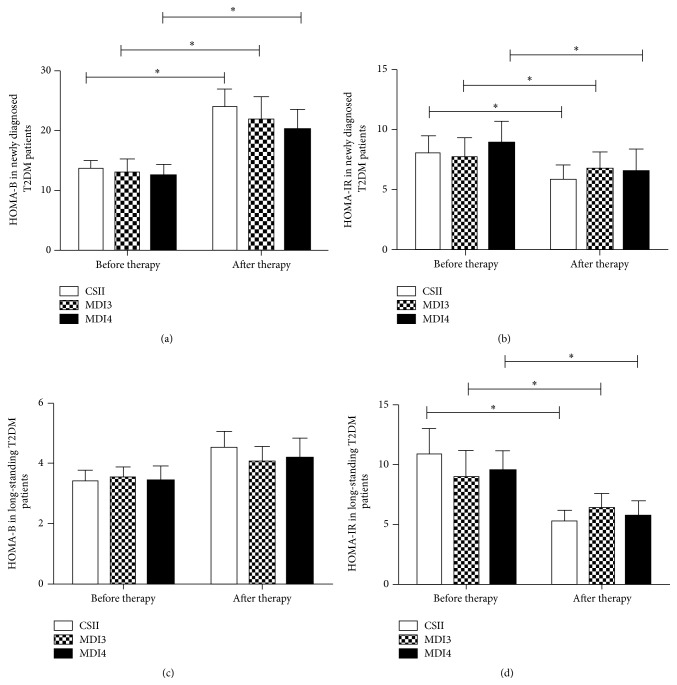
The effect of transient insulin intensive therapy on *β*-cell function and insulin resistance.

In newly diagnosed T2DM patients, the HOMA-B and HOMA-IR were similar among the three treatment groups before treatment. After 2-3 weeks of intensive treatment, the HOMA-B was significantly increased in the newly diagnosed T2DM patients in both CSII and MDI treatments (*P* < 0.05) (Figure 1(a)), accompanied by the improvement in insulin resistance (*P* < 0.05) (Figure 1(b)). Similarly, in long-standing T2DM patients, the HOMA-IR significantly improved after CSII and MDI therapy (*P* < 0.05) (Figure 1(c)). However, the HOMA-B was not dramatically increased (*P* > 0.05), even in patients treated with CSII therapy group (Figure 1(d)).

## 4. Discussion

We have conducted a prospective study on a relatively large number of patients and demonstrated using CGMS that glargine based MDI4 provided better control with less blood glucose fluctuations compared to aspart 30-based MDI3 in long-standing T2DM patients. We also confirmed that treatment with CSII provided a greater improvement of blood glucose fluctuations than either glargine based MDI4 or glargine based MDI3 in newly diagnosed T2DM or long-standing T2DM patients.

CSII and MDI are commonly used forms of intensive insulin therapies. CSII provide precise insulin delivery throughout the day and simulates the function of the islet cells more closely. Use of CSII therapy is now regarded as a safe and valuable alternative in patients with newly diagnosed T2DM. Two to three weeks of early CSII therapy in patients with newly diagnosed T2DM in Chinese population achieved prolonged glycaemic remission, as well as recovery and maintenance of *β*-cell function compared with treatment with oral hypoglycemic agents [8]. Insulin replacement could achieve optimum glycaemic control for 1 year, which might attribute to the increase in acute insulin response, the improvement of qualitative insulin secretion, the reduction in glucotoxicity, and the amelioration of the lipid profile [2, 3, 5, 8]. Furthermore, the early restoration of *β*-cell function and amelioration of insulin resistance might alter the natural history of T2DM [8, 13]. OpT2mise study revealed that, for patients with poorly controlled T2DM, despite using multiple daily injections of insulin, pump treatment can be considered as a safe and valuable treatment option [7]. OpT2mise study enrolled patients from Canada, Europe, Israel, South Africa, and USA. They found that the mean HbA1c of patients in CSII group decreased by −0.7% compared with that in MDI group at 6 months, accompanied by improved blood glucose fluctuations measured by CGM [7].

The goals of intensive therapy can also be achieved by MDI. MDIs are three or more injections daily with intermediate and long-acting insulin. The remission rates after 1 year were significantly higher in the MDI groups than in the oral hypoglycemic agents group [8]. In contrast, study indicated that CSII was superior to MDI with four injections daily in improving HbA1c and postmeal glucose AUC [14].

However, near-normal glucose control is more difficult to achieve, partly because of the limitations of the glycemic profile obtained from intermittent fingerpricks [15]. The intermittent finger pricks were included in a total of three fasting capillary blood glucose monitoring and capillary blood glucose monitoring tests 2 h after each of three meals [8]. Thus, 24 h blood glycemic excursions are undoubtedly missed by these point-to-point glimpses of blood glucose. CGM provides a unique opportunity to examine the 24 h glucose excursions in T2DM when patients achieved euglycemic control.

In the present pilot study, we expected to see a better improvement of blood glucose fluctuations in CSII group compared with MDI group. It is now believed that 2 h glucose concentration may be a better predictor for cardiovascular disease in patients with onset T2DM [16]. Large glucose fluctuations may cause the overproduction of superoxide by the mitochondrial electron-transport chain, which induces a subsequent nitrosative stress [18]. Our data showed a remarkable improved MAGE with CSII therapy compared to MDIs therapy in newly diagnosed T2DM patients. In addition, there was no difference between MDI3 and MDI4 therapy in improvement of MAGE. In contrast, in long-standing T2DM patients, there was better improvement of MAGE in CSII and MDI4 therapy compared to MDI3 therapy. Consistent with this finding, CSII and MDI4 therapy also decreased oxidation stress and inflammation markers in long-standing T2DM patients. It has been demonstrated that repeated fluctuations of glucose increased circulating levels of inflammatory cytokines compared with sustained hyperglycemia [19]. Daniele et al. demonstrated that the inflammatory score, an integrated quantification of TNF-*α*, IL-6, monocyte chemoattractant protein-1, fractalkine, osteopontin, and APN, is increased in patients with T2DM and correlated with hyperglycemia [12].

In addition, our data showed that MDI3 therapy achieved similar improvement of glucose fluctuations either in newly diagnosed T2DM patients or in long-standing T2DM patients. We also did not observe the differences in the incremental AUC (glucose > 10 mmol/L) or the incremental AUC (glucose < 3.9 mmol/L) either in newly diagnosed T2DM patients or in long-standing T2DM patients treated with MDI4 therapy. However, newly diagnosed T2DM patients treated with MDI4 therapy achieved increased improvement of MAGE compared with those in long-standing T2DM patients (4.64 ± 2.21 versus 5.16 ± 1.98 mmol/L, *P* < 0.05), as well as the increasing tendency of the time spent in normal glycaemia (glucose < 10 mmol/L and >3.9 mmol/L) (85 ± 15% versus 73 ± 21%, *P* > 0.05). We could infer that the reason for the differences might partially account the declined *β*-cell function in long-standing T2DM patients compared with new diagnosed T2DM patients (Figures 1(a) and 1(c)). Very recently, Jia et al. indicated that intensive premixed insulin therapy (thrice daily) could further decrease HbA1c level in Asian patients with T2DM who were treated with premixed insulin (twice daily) previously [20]. Our data showed that intensive premixed insulin therapy (thrice daily) could achieve improvement of MAGE in long-standing T2DM patients, which might contribute to the decline of HbA1c level in patients with T2DM treated with intensive premixed insulin therapy [20]. In addition, we also found that long-standing T2DM patients treated with MDI4 therapy achieved greater improvement of MAGE compared with those of MDI3 therapy. A possible explanation might be that MDI4 could more closely mimic physiological insulin secretion compared with MDI3 therapy. However, we have no data for glargine based MDI4 to know if it had favourable outcomes on blood glucose fluctuations control in patients with long-standing T2DM.

Our study has several limitations. First, the study period was 4 years long, from February, 2010, to 24 December, 2014, so the group was heterogeneous. Second, we did not measure the late-phase insulin secretion of *β*-cell. Preserved late-phase insulin secretion might be the key factor in the identification of the patients with established T2DM who can benefit from CSII therapy [6]. Furthermore, the decline in HOMA-IR might partially account the elimination of the deleterious effects of hyperglycemia and the better improvement of blood glucose fluctuations. However, our data could not answer the mechanisms which underline the phenomena. We have now addressed this as another limitation.

In conclusion, CSII results in favourable outcomes on blood glucose fluctuations control either in newly diagnosed patients with T2DM or in patients with long-standing T2DM compared with MDI therapy. In addition, our data suggested that glargine based MDI4 could be considered as a practicable treatment option, if CSII therapy was not available.

## Figures and Tables

**Table 1:** Baseline characteristics of newly diagnosed and long-standing T2DM patients.

	CSII group	MDI3 group	MDI4 group
N	*n* = 39	*n* = 38	*n* = 39
L	*n* = 43	*n* = 41	*n* = 43
Age (years)			
N	55.10 ± 10.02	52.95 ± 9.63	52.56 ± 9.97
L	59.68 ± 8.22	57.67 ± 10.82	58.89 ± 11.85
Men			
N	19 (49%)	18 (47%)	19 (49%)
L	25 (58%)	21 (51%)	23 (53%)
Duration of diabetes (years)			
N	No	No	No
L	12.34 ± 2.07	11.33 ± 1.14	13.28 ± 2.54
Body mass index (kg/m^2^)			
N	24.55 ± 2.90	25.72 ± 3.62	25.03 ± 2.84
L	24.73 ± 3.43	25.17 ± 3.29	24.66 ± 2.84
HbA1c (%)			
N	9.65 ± 1.81	9.99 ± 1.75	10.13 ± 1.93
L	8.38 ± 1.68	8.18 ± 1.58	8.66 ± 1.73
Systolic blood pressure (mm Hg)			
N	126.77 ± 12.55	127.05 ± 16.44	131.03 ± 15.56
L	129.12 ± 12.76	127.41 ± 15.28	132.33 ± 11.17
Diastolic blood pressure (mm Hg)			
N	81.10 ± 9.84	81.64 ± 10.15	81.25 ± 6.78
L	82.72 ± 10.46	83.67 ± 11.41	83.06 ± 11.10
Fasting plasma glucose (mmol/L)			
N	10.47 ± 2.62	11.07 ± 3.01	11.24 ± 2.94
L	9.32 ± 2.22	8.66 ± 2.75	9.56 ± 2.35
Fasting plasma insulin (mU/L)			
N	5.58 ± 2.95	7.40 ± 8.84	6.79 ± 2.91
L	5.97 ± 2.86	6.01 ± 4.19	6.11 ± 3.15
Fasting plasma C-peptide (pmol/L)			
N	2.23 ± 0.77	1.98 ± 1.07	2.36 ± 1.12
L	1.94 ± 0.60	2.41 ± 1.11	2.02 ± 0.62

Data are mean (SD) or *n* (%). N: newly diagnosed T2DM patients group; L: long-standing T2DM patients group.

**Table 2:** Oxidative stress and inflammatory profile of newly diagnosed and long-standing T2DM patients.

	CSII N group	MDI3 N group	MDI4 N group
8-PGF_2*α*_ (pg/mL)			
N			
Before therapy	8.11 ± 2.77	9.46 ± 0.89	10.15 ± 2.34
After therapy	6.12 ± 2.66^*∗∗*^	7.00 ± 1.93^*∗∗*^	6.70 ± 2.53^*∗∗*^
L			
Before therapy	8.07 ± 2.89	8.58 ± 3.14	8.46 ± 1.75
After therapy	4.10 ± 2.93^*∗∗*^	5.17 ± 3.88^*∗∗*^	4.16 ± 2.10^*∗∗*^
TNF-*α* (pg/mL)			
N			
Before therapy	19.59 ± 4.56	18.01 ± 7.41	17.11 ± 6.32
After therapy	9.08 ± 5.14^*∗∗*^	10.26 ± 3.33^*∗*^	8.12 ± 5.00^*∗∗*^
L			
Before therapy	13.46 ± 2.99	14.53 ± 3.23	11.65 ± 4.58
After therapy	6.08 ± 1.14^*∗*†^	8.70 ± 2.34^*∗*^	6.25 ± 2.37^*∗∗*†^
IL-6 (pg/mL)			
N			
Before therapy	4.51 ± 1.87	5.08 ± 1.12	6.08 ± 4.52
After therapy	2.17 ± 1.43^*∗*^	2.60 ± 0.78^*∗*^	2.90 ± 1.84^*∗*^
L			
Before therapy	3.66 ± 2.19	4.12 ± 1.88	3.73 ± 2.00
After therapy	2.62 ± 2.16^*∗∗*†^	4.32 ± 1.45^*∗*^	3.01 ± 2.55^*∗∗*†^

Data are mean ± (SD). ^*∗*^ *P* < 0.05, ^*∗∗*^ *P* < 0.01 versus the same item before therapy; ^†^ *P* < 0.05 versus MDI3 N group after therapy. N: newly diagnosed T2DM patients group; L: long-standing T2DM patients group.

**Table 3:** Blood glucose fluctuations of newly diagnosed T2DM patients.

	CSII N group	MDI3 N group	MDI4 N group
24 h MBG (mmol/L)			
Before therapy	11.55 ± 2.63	12.51 ± 2.73	12.20 ± 2.74
After therapy	7.70 ± 1.79^*∗∗*^	7.55 ± 1.13^*∗∗*^	7.44 ± 1.40^*∗∗*^
MAGE			
Before therapy	7.11 ± 2.87	6.87 ± 2.01	6.31 ± 3.62
After therapy	4.51 ± 1.92^*∗*^	5.05 ± 1.97^*∗*^	4.94 ± 2.21^*∗*^
The time spent (>10 mmol/L) (%)			
Before therapy	62 ± 31	67 ± 29	66 ± 31
After therapy	14 ± 17^*∗∗*^	14 ± 13^*∗∗*^	12 ± 14^*∗∗*^
The time spent in <3.9 mmol/L (%)			
Before therapy	2 ± 4	1.8 ± 5	2.6 ± 6
After therapy	0.32 ± 0.8^*∗∗*^	0.4 ± 0.8^*∗∗*^	0.7 ± 0.3^*∗∗*^
The time spent in normal glycaemia (%)			
Before therapy	38 ± 31	33 ± 29	38 ± 36
After therapy	85 ± 16^*∗∗*^	83 ± 13^*∗∗*^	84 ± 15^*∗∗*^
The AUC (>10 mmol/L) (mmol/L per day)			
Before therapy	2.55 ± 1.99	2.84 ± 2.29	2.81 ± 2.02
After therapy	0.29 ± 0.57^*∗∗*^	0.27 ± 0.37^*∗∗*^	0.27 ± 0.35^*∗∗*^
The AUC for <3.9 mmol/L (mmol/L per day)			
Before therapy	0.02 ± 0.08	0.01 ± 0.05	0.02 ± 0.09
After therapy	0.00 ± 0.00^*∗∗*^	0.00 ± 0.02^*∗∗*^	0.00 ± 0.03^*∗∗*^

Data are mean ± (SD). ^*∗*^ *P* < 0.05, ^*∗∗*^ *P* < 0.01 versus the same item before therapy.

**Table 4:** Blood glucose fluctuations of long-standing T2DM patients.

	CSII N Group	MDI3 N Group	MDI4 N Group
24 h MBG (mmol/L)			
Before therapy	10.80 ± 2.82	10.00 ± 2.60	11.43 ± 3.19
After therapy	7.69 ± 2.24^*∗∗*^	7.65 ± 1.15^*∗∗*^	8.07 ± 1.40^*∗∗*^
MAGE			
Before therapy	6.56 ± 2.77	6.95 ± 2.74	6.48 ± 3.15
After therapy	4.62 ± 2.97^*∗*†^	6.27 ± 1.83^*∗*^	5.16 ± 1.98^*∗*†^
The time spent (>10 mmol/L) (%)			
Before therapy	60 ± 32	65 ± 27	62 ± 36
After therapy	16 ± 17^*∗∗*^	19 ± 10^*∗∗*^	18 ± 15^*∗∗*^
The time spent in <3.9 mmol/L (%)			
Before therapy	3 ± 9	4 ± 5	1 ± 2
After therapy	0.2 ± 0.2^*∗∗*†^	0.4 ± 0.1^*∗∗*^	0.2 ± 0.3^*∗∗*†^
The time spent in normal glycaemia (%)			
Before therapy	39 ± 31	33 ± 29	38 ± 36
After therapy	83 ± 17^*∗∗*†^	73 ± 10^*∗∗*^	79 ± 11^*∗∗*†^
The AUC (>10 mmol/L) (mmol/L per day)			
Before therapy	2.34 ± 2.12	2.77 ± 1.73	2.93 ± 2.50
After therapy	0.25 ± 0.60^*∗∗*^	0.37 ± 0.27^*∗∗*^	0.28 ± 0.09^*∗∗*^
The AUC for <3.9 mmol/L (mmol/L per day)			
Before therapy	0.02 ± 0.08	0.02 ± 0.04	0.06 ± 0.02
After therapy	0.00 ± 0.00^*∗∗*^	0.00 ± 0.00^*∗∗*^	0.00 ± 0.00^*∗∗*^

Data are mean ± (SD). ^*∗*^ *P* < 0.05, ^*∗∗*^ *P* < 0.01 versus the same item before therapy; ^†^ *P* < 0.05 versus MDI3 N group after therapy.
